# Multiple HPV 16 infection with two strains: a possible marker of neoplastic progression

**DOI:** 10.1186/s12885-020-06946-7

**Published:** 2020-05-19

**Authors:** Maria Teresa Bruno, Guido Scalia, Nazario Cassaro, Sara Boemi

**Affiliations:** 1 Department of General Surgery and Medical Surgery Specialties, Gynecological Clinic, University of Catania, Catania, Italy; 2 Department of Biomedical and Biotechnological Sciences, Clinical Virology, University of Catania, Catania, Italy; 3 Gynecological Oncology, Humanitas, Catania, Italy

**Keywords:** HPV infection, Multiple HPV infection, CIN2+

## Abstract

**Background:**

We studied the cases of single and multiple HPV infection and analyzed the correlation with negative cases, and preneoplastic and neoplastic lesions of the uterine cervix with the aim of making a contribution to the prognostic factor under discussion.

**Methods:**

Nine hundred nine women undergoing second level screening because they had been positive at cervical cytology were enrolled.

All the patients underwent colposcopy and cervical biopsy with viral genotyping. We divided mHPV infection based on the number of genotypes present: infections with 2 strains, 3 strains, 4 strains and 5 or more strains.

**Statistical analysis:**

The analysis of the data was made using the χ2 test. Contingency tables were created to evaluate the correlation between single, multiple and CIN2+ infections. Values with *p* < 0.05 were considered statistically significant.

**Results:**

The presence of genotype HPV16 in our study was associated with a 12 times greater risk of developing a high-grade lesion, OR = 12.70. The patients with single infections had the highest incidence of CIN2+ (34.1%) with respect to those with multiple infections (10.6%).When we studied in the mHPV infection the prevalence of the combinations between the genotypes, we found that in mHPV16 infections, the combinations HPV16, 18 and HPV16, 31 were the most frequent (55.5%) in CIN3 lesion.

**Conclusions:**

Our results suggest that single HPV infections have a greater risk of developing SCC with respect to multiple infections. Multiple HPV infections are relevant only in the first phase of the lesion (CIN1-CIN2), while they are absent in carcinomas, where infections are of a single genotype. In particular, among multiple infections, HPV16 infection with 2 HR genotypes is associated significantly with CIN2 / CIN3 (21/30) and has 4 times greater risk of developing a high-grade lesion. Thus, it is probable that only specific combinations of HPV (HPV16,18 - HPV 16,31) can be associated with a clinically significant impact, while other combinations can simply be correlated because of a common infection or diagnostic method used. Therefore, multiple HPV16 infections with two high-risk genotypes is a major risk of CIN2/CIN3.

## Background

Human papillomavirus (HPV) Infection of the cervix is a sexually transmitted disease and a significant risk factor for the development of cervical intraepithelial neoplasia (CIN) [1]. However, only a small percentage of women with the infection develop CIN2 + (CIN2, CIN3, SCC). Various risk factors exist leading to cervical carcinoma: viral genotype, age, viral persistence, and a woman immune status of the. All these factors have been well demonstrated by various authors, however, there has recently been a discussion concerning another element: multiple papillomavirus infections (mHPV infections) recognized as risk factors for cervical carcinoma [2].

The prevalence of the various viral genotypes shows considerable differences worldwide [3] and the increase of international migratory flows inevitably lead to a continual and constant increase of the diversification of the viral genotypes present in Italy.

The clinical, virological and epidemiological significance of mHPV is still not clear: some reports in the literature suggest a possible role in the development and progression of cervical neoplasia, while other studies [4] show how the risk of development of precancerous lesions and invasive tumors in women with more than one type of HPV are not more than those of women with an infection from a single genotype. When more than one type of HPV can be found, it is difficult to assign the causality of a single lesion CIN to a particular type of HPV. One approach is to use the association between the persistence of a type of HPV in previous cytological samples and the development of CIN [5] . However, because of the difficulty of attributing the lesion to a particular HPV genotype, it has not yet been demonstrated if the association is given by the simple added risk or the synergic interaction between the various types of HPV [6]. The studies of Liaw [7] are important as they associate HPV16 infection with an increased risk of contracting multiple infections, while the studio of Chaturvedi [8] reported that the A9 group, made up of genotypes 16–31–33-35-52 and 58, is significatively less involved in multiple infections with respect to genotypes belonging to other groups; it seems that the types of HPV involved in multiple infections do not follow a particular logic of combination. In the light of these considerations, we studied the cases of infection by single and multiple HPV and analyzed the correlation with negative cases and preneoplastic and neoplastic lesions of the uterine cervix with the aim of contributing to the prognostic factor in discussion.

## Methods

From January 2014 to November 2017, at the outpatients’ clinic for colposcopy of the University of Catania, Italy, 909 women undergoing second level screening because they had been positive at cervical cytology were enrolled. The data were kept in a database at the University Hospital of Catania, Italy for a retrospective study.

The study was carried out in conformity with the Declaration of Helsinki, 1975.

The inclusion criteria were the following:

- women positive at cytology for lesions correlated to HPV
- women with preneoplastic or neoplastic lesions of the uterine cervix diagnosed by a histological exam

Women who were pregnant, immuno-depressed or with infections caused by human immunodeficiency virus (seropositive) were not enrolled. The age of the patients was between 16 and 59 years, mean 32 ± 8,3 years. All the patients underwent colposcopy and cervical biopsy with viral genotyping. The histological evaluation was carried out on biopsy samples from colposcopy and/or cone samples from LEEP. Histology was diagnosed according to the OMS classification as CIN2 + for all the cases of lesions CIN2, CIN3 and SCC.

In agreement with the LAST Project, we treated CIN1 as a simple viral lesion and we considered only CIN2 + lesions as true preneoplastic lesions.

Detection of viral DNA (HPV test) was carried out using Polymerase Chain Reaction (PCR) after the extraction of the cervical sample using Thin-prep. Automatic DNA extraction was carried out using the Nucli Senseasy MAG system (bioMérieux SA, Marcy l’Etoile, France) following the HPV 1.1 protocol of the manufacturer. HPV DNA amplification was made by HPV-HS Bio (AB Analitica s.r.l, Padova, Italia) nested-PCR for the detection of the HPV-DNA sequence inside ORF L1, according to the manufacturer’s instructions. HPV typing was carried out with a probe specific for the most frequent types of HPV (type HPV, AB Analitica s.r.l., Padova, Italy). HPV typing identified 11 LR genotypes (6, 11, 40, 42, 43, 44, 54, 61, 70, 72, 81) and 18 HR genotypes (16, 18, 26, 31, 33, 35, 39, 45, 51, 52, 53,56, 58, 59, 66, 68, 73, 82). The positive samples at nested-PCR but negative in reverse line blot for any of these types were considered as undetermined HP. The cervical swab for the HPV test was taken from the endocervical canal and the transformation zone. We divided the multiple infections based on the number of genotypes present: infections with 2 strains, 3 strains, 4 strains and 5 or more strains.

### Statistical analysis

The statistical analysis of the data was carried out with the software package SPSS 15.0 (SPSS Inc.; Chicago, IL, USA). The analysis of the data was made using the χ2 test and, if necessary, Fisher’s exact test was used to calculate the statistical significance (*p* value) of the difference between groups. Contingency tables were created to evaluate the correlation between single, multiple and CIN2+ infections. Values with *p* < 0.05 were considered statistically significant.

## Results

Viral genotyping gave a positive result for single infections in 360 patients, 387 patients were positive for multiple infections, 127 women were negative for the viral genotypes present in the kit used. Thirty-six samples analyzed, from among all the samples, were inadequate; these patients and the negative women were excluded from the study group.

At the histological exam, carried out after biopsy, 458/747 patients (61.3%) presented a low-grade preneoplastic lesion (CIN 1); 64 women (8.6%) had a moderate-grade lesion (CIN 2), 105 women (14%) had a sever-grade lesion (CIN 3); and 5 women (0.7%) had squamosa carcinomas (SCC). Finally, 115 (15.4%) patients had a negative histological diagnosis (Table 1). In total we had 174 CIN2+ (23.3%) lesions.

**Table 1:** Prevalence of histological findings and viral genotyping results in the study group

HPV infection	Negativi	CIN1	CIN2	CIN3	SCC
Single infection 360 (48,2%)	56 (48,6%)	181 (39,5%)	36 (56,2%)	82 (78%)	5 (100%)
Multiple infection 387 (51,8%)	59 (51,3%)	277 (60,4%)	28 (43,7%)	23 (22%)	0
Totale 747	115 (15,4%)	458 (61,4%)	64 (8.6%)	105 (13.9%)	5 (0,7%)

### Single HPV infection

There were 360 (48.2%) single infections in our group with 123 lesions (34.1%) CIN2+. The typology of lesions with the highest percentage of a single infection was SCC with 100%, followed by CIN3 with 78%, CIN2 with 56.2% and finally negative and CIN1 with 48.6 and 39.5%, respectively. The genotype most frequently found was genotype 16, present in 142/360 cases (39.4%), and was responsible for 76.4% (94/123) of the cases of CIN2+, 21.6% of the cases of CIN1 and 16% of negatives (Table 2).

**Table 2:** Percentage of single infection in the study group with different cervical lesion grades

HPV		Negative	CIN1	CIN2	CIN3	CSS
HR	HPV16	9 (28,1)	39 (31,4%)	24 (67,5%)	66 (80,5%)	4 (80%)
	other	23 (71,8%)	85 (68,5%)	12 (32,4%)	16 (19,5%)	1 (20%)
LR		24	57	0	0	0
		56	181	36	82	5

In the present study the patients with a single infection from HPV 16 had the highest incidence (76.4%) of CIN2+ with respect to the other HR genotypes (23.5%).

In particular, the presence of genotype HPV16 in our study was associated with a 12 times greater risk of developing a high-grade lesion (CIN2+), OR = 12.70; (IC 95% = 7.52–21.42). After genotype 16, the genotype most represented was 31 with 36 cases, of which 11 CIN2+, while the genotypes 18 and 45 were poorly represented with 17 cases of which 8 CIN2+ and 6 cases of 1 CIN3. Other HR HPV found were 33 with 8 cases, of which 2 CIN3, 35 with 4 cases, of which 2 CIN3 and 52 and 58 both with 1 case of CIN2. (Table 3). It should be noted that the most frequent genotypes were 16, 31, 33, 35, 52 and 58 belonging to the same group alfa9.

**Table 3:** Prevalence of histological findings and HR HPV genotypes in the study group

HPV infection	Negative	CIN1	CIN2	CIN3	SCC
**HRHPV Single Infection**	**32 (11,4%)**	**124 (43%)**	**36 (12,9%)**	**82 (29,3%)**	**5 (1,8%)**
**HPV16**	9	39	24	66	4
**HPV18**	2	7	4	4	0
**HPV31**	2	23	3	7	0
**HPV45**	2	3	0	1	0
**HPV33**	1	5	0	2	0
**HPV35**	0	2	0	2	0
**HPV51**	0	6	3	0	1
**HPV52**	1	4	1	0	0
**HPV53**	1	9	0	0	0
**HPV56**	4	4	0	0	0
**HPV58**	1	8	1	0	0
**HPV59**	2	4	0	0	0
**HPV67**	0	1	0	0	0
**HPV68**	1	3	0	0	0
**HPV70**	1	3	0	0	0
**HPV73**	3	3	0	0	0
**HPV81**	1	2	0	0	0
**HPV82**	1	0	0	0	0

Particular importance needs to be given to genotype 51 that demonstrated with 3 cases of CIN2 and 1 squamous carcinoma to have an oncogenic capacity and also genotype 35, responsible for 2 cases of CIN3. Both 51 and 35 genotypes have not been assembled among the genotypes of the nonovalent vaccines.

### Multiple HPV infections

**Fig. 1:**
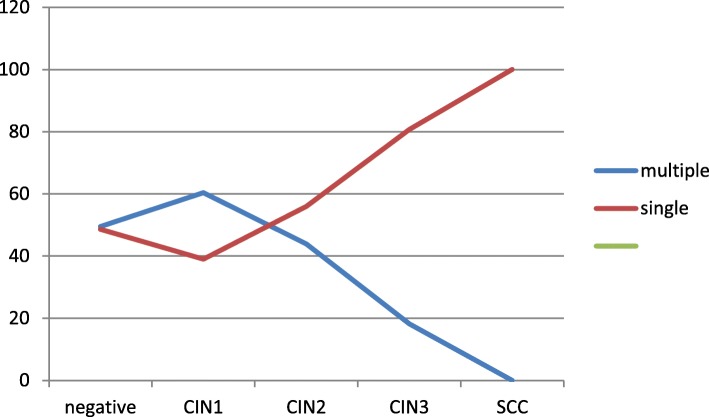
Prevalence of single and multiple HPV infection and lesion grade

There were 387 (51.8%) multiple infections in our study with an incidence of high-grade lesions of 10.6% (41/387). The typology of the lesion with the highest percentage of multiple infections was CIN1 with a frequency of 60.4% and the negative cases were 50.4%. The percentage decreases more in CIN2 (43.7%) and progressively in CIN3 (22.1%), arriving at zero in carcinomas, characterized exclusively by single infections (Fig. 1). We had no cases of SCC with multiple genotypes.

The four most diffused HPV genotypes were HPV 16, 51, 59 and 31. Also in multiple infections the most frequent genotype was 16 (151/387) with a prevalence of 74.5% (38/51), this was significatively higher in patients with CIN2/CIN3, OR = 5.77 (IC 95% = 2.95–11.26). We studied in particular genotype 16 (Table 4). We analyzed the difference between infections of single and multiple types with HPV16 and the different impact that both the infections have on severe dysplasia and carcinomas: the patients with single infections had the highest incidence of CIN2+ (70.1%) with respect to those with multiple infections (29,3%).

**Table 4:** Percentage of multiple infection in the study group with different cervical lesion grades

HPV	Negative	CIN1	CIN2	CIN3	CSS
HPV16 othersHR+LH	20 (33,9%)	93 (33,5%)	16 (58,6%)	22 (95,6%)	0
	39 (66,1%)	184 (66,5%)	12 (32,4%)	1 (4,3%)	0
	59	277	28	23	0

OR of the single infections from HPV16 associated with CIN2+ was greater than the OR of multiple HPV16 infections (Table 5).

**Table 5:** OR for CIN2+ in single and multiple HPV16 infection

	OR	95% CL	P
Single	12,7	(7,52-21,42)	0.0005
Multiple	5,77	(2,95- 11,26)	0.0005

We divided multiple infection by the number of genotypes present (strains) into 2 strains, 3 strains, 4 strains, 5 strains or more. We found that increasing the histological grade of the lesion, decreases the number of HR genotypes present; in fact, CIN3 lesion has two high-risk genotypes in 82.6% of cases (19/23). the remaining 4 cases (4/23) had three genotypes. We did not have any cases of CIN3 lesion with 4 or 5 genotypes.

We correlated genotype 16 with the number of strains and the histological diagnosis (Table 6). CIN 3 other than representing 82.6% of two high-risk genotypes, one of these genotypes is almost always genotype 16 (22/23).

**Table 6:** Correlation between HPV16, number of strains and histologic diagnosis

Strains n°	Negative		CIN1		CIN2		CIN3	
	HPV16	others	HPV16	others	HPV16	others	HPV16	others
**2**	**10**	**24**	**39**	**110**	**12**	**9**	**18**	**1**
**3**	**3**	**9**	**23**	**47**	**2**	**2**	**4**	**0**
**4**	**6**	**4**	**21**	**16**	**1**	**1**	**0**	**0**
**5**	**1**	**1**	**10**	**8**	**2**	**0**	**0**	**0**

**Fig. 2:**
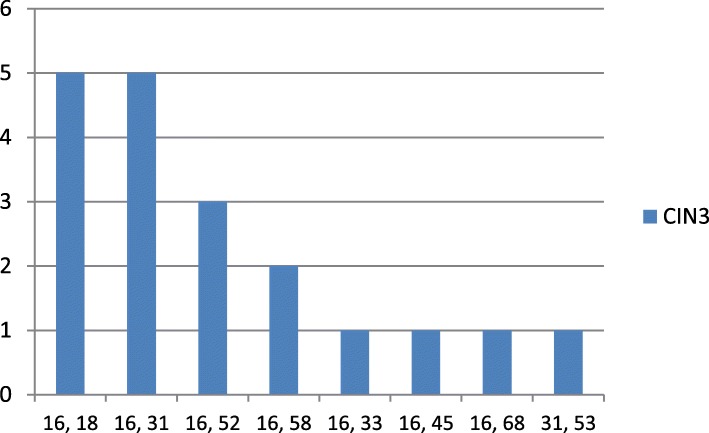
Multiple HPV infection, prevalence of the combination between the genotypes in CIN3

When we studied the prevalence of the combinations between the genotypes, we found that the combinations HPV16, 18 and HPV16, 31 were the most frequent (55.5%) in CIN3 (Fig. 2).

From the OR analysis, multiple HPV16 infections with 2 high-risk genotypes, with respect to infections with 3 or more genotypes, were significant with an OR = 3.92 (IC% 1.70–9.03) for CIN2+.

## Discussion

To date, only a few studies have reported a relationship between mHPV infections and SCC, and the results have not been conclusive. Some studies have suggested a possible role of mHPV in the development of SCC [8–10], while other studies have reported that the risk of SCC in women infected by mHPV was not greater than that with single type infections [11, 12].

Fife KH [10] found, an average of 3 different types of HPV in cervical dysplasia, with respect to the single type of HPV in samples with normal cytology, supporting a possible role of more types of HPV infections in the development of cervical dysplasia; Becker [13] studied the presence of multiple papillomavirus infections in immune-competent women, demonstrating that these infections are associated with a greater risk of dysplasia, second only to positivity for HPV16. It has also been hypothesized that various types of HPV act co-operatively in neoplastic transformations. Rousseau [14] on the other hand, suggested that the persistence of papillomavirus infection can be independent from the presence of more viral genotypes. Cuschieri [12] found an elevated prevalence of more high-risk HPV types in all the grades of cervical neoplasia stressing the lack of a cooperative relationship between the couples of cancerogeni or groups of high-risk HPV types.

Rolòn [15] did not find a significatively higher risk of carcinoma in women with multiple HPV types infections with respect to those affected by a single viral genotype.

The relevance of multiple infections in the last few years has shown an increase in cases thanks to improved PCR sensitivity. In studies published in the 1990s, multiple infections were only 4%, in more recent studies multiple infections have reached 15.7%. The only exception are the women affected by HIV who often present with multiple infections (80%) [16], in these women multiple infections are often associated with CIN2+, suggesting a possible role of systemic immunodeficiency, which would favor the acquisition, as a determining factor for the development of multiple infections and Weiland found multiple infections in HIV positive women a prognostic factor of CIN2+ lesions.

This changes in immuno-competent women in whom the prevalence of mHPV varies from 24.8% to 52,6% between all HPV positive women [17].

In our study, the prevalence of multiple infections was 51,8%, in particular 60.4% in patients with CIN1, to 22% in those with CIN3; the prevalence of multiple infections in the group of patients with a negative histologic exam was 51,3%; we had no SCC cases.

The prevalence of multiple infections is high, thus, in negatives and cases of slight dysplasia, CIN2 and CIN3 decreased until it was absent in neoplasia, while the prevalence of infections from a single genotype was lower among negatives and CIN1, rising again in severe dysplasia and carcinoma (Fig. 1).

Studying the prevalence curve of multiple infections underlines the lack of an oncogenic relation of multiple infections in the genesis of preneoplastic lesions and carcinoma and favors the presence of more transitory sexually transmitted infections.

In most cases, HR genotypes and LH genotypes co-exist giving rise to the origin of transitory infections that rapidly clear up. In fact, coinfections are relevant only in the first phase of the lesion (CIN 1 and CIN2), while they are absent in carcinomas in which there are only single lesions. Moreover, we found that increasing grades of dysplasia decreases the number of the genotypes present, in fact, transversal analysis of the our data showed that the detection of CIN2-CIN3 in the presence of multiple HPV infections seems to be significantly correlated to the presence of infections due to two strains of high-risk genotypes (in particular, the couples 16, 31 and 16, 18). In about 95% of invasive cervical tumors positive for HPV, and in all our cases of SCC, only 1 type of HPV was found [18–20]. This lends support to the concept of a clonal development of invasive neoplasia, deriving from a persistent infection with a single HPV type [21]. In CIN3 and SCC, a monoclonal line is developed from an infected cell with a single high-risk HPV, with the consequence of finding few multiple infections in high-grade lesions and only single infections in carcinoma [22]. An important contribution in the study of the relationship between HPV and CIN is status given by Wim Quint [23] who, using LCM-PCR was able to accurately determine the HPV types in normal and abnormal epithelium of cervical biopsies colored with H & E and p16 demonstrating that most women with more cervical HPV infections have only 1 type of HPV per lesion. In the same cervix different grade of CIN can co-exist and each lesion is from a different HPV. The most important conclusion is that each type of HPV seems to be associated with an independent CIN lesion and that in multiple infections there is no synergic action of more HR genotypes towards carcinogenesis. From a pathogenetic point of view, the data seem to suggest the hypothesis [22] that different cells are infected by different viruses, rather than in the same cell various viral genotypes exist.

## Conclusions

Our results suggest that single HPV infections have a greater risk of developing SCC with respect to multiple infections. Multiple HPV infections are relevant only in the first phase of the lesion (CIN1-CIN2), while they are absent in carcinomas, where infections are of a single genotype. Also in our study the genotype 16 is the one with the highest oncogenic risk, genotype 16 is significatively correlated with high-grade lesions so much so that some authors consider reserving dedicated guide-lines for HPV16 positive patients [24].

In particular, among multiple infections, HPV16 infection with 2 HR genotypes is associated significantly with CIN2 / CIN3 (21/30) and has 4 times greater risk of developing a high-grade lesion. Thus, it is probable that only specific combinations of HPV (HPV16,18 - HPV 16,31) can be associated with a clinically significant impact, while other combinations can simply be correlated because of a common infection or diagnostic method used. Therefore, multiple HPV16 infections with two high-risk genotypes is a major risk of CIN2/CIN3.

The limits of our study are connected to the samples studied in as much as the women in our study came from a second level center.

Further clinical studies are needed to determine the mechanism of these mHPV infections and if the clinical results are correlated with a particular combination of types of HPV.

## Data Availability

The datasets used and/or analyzed during the current study are available from the corresponding author on. request.

## Abbreviations

HPV: Human papillomavirus
CIN2 +: All the cases of CIN2, CIN3, SCC lesion
hrHPV: High riskHPV
SCC: Squamous cervical carcinoma
mHPV: Multiple HPV infection
LCM-PCR: Laser capture micro-dissection PCR
